# Accelerometer-Measured Inpatient Physical Activity and Associated Outcomes After Major Abdominal Surgery: Systematic Review

**DOI:** 10.2196/46629

**Published:** 2023-05-15

**Authors:** Mikita Fuchita, Kyle J Ridgeway, Clinton Kimzey, Edward L Melanson, Ana Fernandez-Bustamante

**Affiliations:** 1 Department of Anesthesiology University of Colorado Anschutz Medical Campus Aurora, CO United States; 2 Department of Inpatient Rehabilitation Therapy University of Colorado Hospital University of Colorado Health Aurora, CO United States; 3 Department of Physical Medicine and Rehabilitation University of Colorado Anschutz Medical Campus Aurora, CO United States; 4 University of Colorado School of Medicine University of Colorado Anschutz Medical Campus Aurora, CO United States; 5 Department of Medicine Division of Endocrinology and Metabolism University of Colorado Anschutz Medical Campus Aurora, CO United States; 6 Department of Medicine Division of Geriatric Medicine University of Colorado Anschutz Medical Campus Aurora, CO United States; 7 Eastern Colorado Veterans Affairs Geriatric Research, Education and Clinical Center US Department of Veterans Affairs Aurora, CO United States

**Keywords:** abdominal surgery, accelerometry, early mobilization, physical activity, postoperative care, wearable

## Abstract

**Background:**

It remains unclear how inpatient physical activity after major abdominal surgery affects outcomes. Accelerometer research may provide further evidence for postoperative mobilization.

**Objective:**

We aimed to summarize the current literature evaluating the impact of accelerometer-measured postoperative physical activity on outcomes after major abdominal surgery.

**Methods:**

We searched PubMed and Google Scholar in October 2021 to conduct a systematic review. Studies were included if they used accelerometers to measure inpatient physical behaviors immediately after major abdominal surgery, defined as any nonobstetric procedures performed under general anesthesia requiring hospital admission. Studies were eligible only if they evaluated the effects of physical activity on postoperative outcomes such as postoperative complications, return of gastrointestinal function, hospital length of stay, discharge destination, and readmissions. We excluded studies involving participants aged <18 years. Risk of bias was assessed using the risk-of-bias assessment tool for nonrandomized studies (RoBANS) for observational studies and the revised Cochrane risk-of-bias tool for randomized trials (RoB 2) for randomized controlled trials (RCTs). Findings were summarized by qualitative synthesis.

**Results:**

We identified 15 studies. Risk of bias was high in 14 (93%) of the 15 studies. Most of the studies (11/15, 73%) had sample sizes of <100. Of the 15 studies, 13 (87%) included the general surgery population, 1 (7%) was a study of patients who had undergone gynecologic surgery, and 1 (7%) included a mixed (abdominal, thoracic, gynecologic, and orthopedic) surgical population. Of the 15 studies, 12 (80%) used consumer-grade accelerometers to measure physical behaviors. Step count was the most commonly reported physical activity outcome (12/15, 80%). In the observational studies (9/15, 60%), increased physical activity during the immediate postoperative period was associated with earlier return of gastrointestinal function, fewer surgical and pulmonary complications, shorter hospital length of stay, and fewer readmissions. In the RCTs (6/15, 40%), only 1 (17%) of the 6 studies demonstrated improved outcomes (shorter time to flatus and hospital length of stay) when a mobility-enhancing intervention was compared with usual care. Notably, mobility-enhancing interventions used in 4 (67%) of the 6 RCTs did not result in increased postoperative physical activity.

**Conclusions:**

Although observational studies show strong associations between postoperative physical activity and outcomes after major abdominal surgery, RCTs have not proved the benefit of mobility-enhancing interventions compared with usual care. The overall risk of bias was high, and we could not synthesize specific recommendations for postoperative mobilization. Future research would benefit from improving study design, increasing methodologic rigor, and measuring physical behaviors beyond step counts to understand the impact of postoperative mobilization on outcomes after major abdominal surgery.

## Introduction

### Background

Clinicians used to prescribe strict bed rest for 2 to 3 weeks after abdominal surgery until pioneers such as Leithauser started challenging this dogma in the 1940s [1]. Through a series of case studies, these pioneers reported that immobility caused harm and that early mobilization was safe and feasible [2-4]. In 2005, the Enhanced Recovery After Surgery (ERAS) Society published its first perioperative guidelines for patients undergoing colorectal surgery [5] and promoted the uptake of early mobilization efforts by clinicians. The guidelines recommended that patients spend 2 hours out of bed on the day of surgery and 6 hours per day out of bed until discharge [5]. Today, ERAS guidelines have expanded to >20 adult specialties, all of them describing early mobilization as a vital component of postoperative care [6].

Despite the widespread acceptance of the ERAS guidelines, the recommendations on postoperative mobilization are built on expert consensus with little to no data supporting the specific mobility goals [7,8]. Early mobilization remains poorly defined in the literature [7-9], and protocols vary substantially between institutions and studies [8,9]. Hence, optimal methods to achieve early mobilization and the impact of specific physical activity components (such as timing, type, duration, frequency, and intensity) [10,11] on postoperative outcomes are still unknown [7-9].

### Objectives

Accelerometers have gained popularity as consumer- and research-grade activity-tracking devices [12]. Their ability to quantitatively measure and summarize physical behaviors has attracted many researchers, and, as a result, the number of publications using accelerometers has grown exponentially in recent years [13]. In this systematic review, we aimed to summarize the current literature on accelerometer-measured postoperative physical activity in the acute inpatient setting and its impact on clinical outcomes after major abdominal surgery.

## Methods

### Overview

We first searched PROSPERO [14] to verify the absence of existing or ongoing research on this topic. We then outlined a written protocol (not registered) according to the PRISMA (Preferred Reporting Items for Systematic Reviews and Meta-Analyses) statement [15] before conducting the literature search. We adhered to our protocol and the PRISMA statement throughout the review process (Multimedia Appendix 1).

### Search Strategy

We searched PubMed and Google Scholar using comprehensive search strategies developed with assistance from an institutional librarian. The search strategy included the Medical Subject Headings (MeSH) terms “postoperative period,” “postoperative care,” “accelerometry,” “wearable electronic devices,” “fitness trackers,” and their related terms (Multimedia Appendix 2). The database included all publications up to October 14, 2021. For Google Scholar, we screened the first 100 articles as described previously [16]. Reference lists of related studies were also used to identify relevant articles.

### Inclusion and Exclusion Criteria

We included studies that used accelerometers to measure physical activity during the hospital stay immediately after major abdominal surgery. We defined major abdominal surgery as any nonobstetric procedures performed under general anesthesia requiring hospital admission. Both open and laparoscopic surgeries were eligible. Studies were eligible only if they evaluated the effects of physical activity on postoperative outcomes. Outcomes of interest included postoperative complications, return of gastrointestinal function, hospital length of stay, discharge destination, and readmissions.

We excluded studies if they (1) involved participants aged <18 years; (2) did not include physical activity measurements during the acute inpatient period; (3) only reported descriptive analysis of physical activity measures without evaluating their impact on clinical outcomes; or (4) were case reports, study protocols, or conference abstracts. Review articles were used to search for additional articles not captured by the database search.

### Study Selection

After the literature search, we used Covidence systematic review software (Veritas Health Innovation Ltd) to facilitate study selection, data extraction, and quality assessment. First, 2 independent reviewers (MF and KJR) screened titles and abstracts of all articles identified from the database searches and the studies identified from the reference lists. Studies were excluded if they were not relevant (eg, nonabdominal surgery and use of accelerometers for purposes other than physical activity measurements). The included studies underwent a full-text review by 2 independent reviewers (MF and KJR) using the aforementioned inclusion and exclusion criteria. Any disagreements between the reviewers for each of these steps were resolved by a third reviewer (AFB).

### Data Extraction

Data extraction was performed by a single investigator (MF). The data collection form contained the following variables: study design, type of surgery, number of participants, patient characteristics, descriptions of interventions (if applicable), device name, device setup (eg, sampling rate, filter, and epoch), device wear location, data collection period, reported measures of physical activity (including but not limited to step count, postural transition, activity duration, time-to-mobilization events, and activity trend over time), and clinical outcomes. We extrapolated the duration of device wear in the hospital based on the methods described in each study.

### Quality Assessment

We used the risk-of-bias assessment tool for nonrandomized studies (RoBANS) [17] to assess the risk of bias in observational studies and the revised Cochrane risk-of-bias tool for randomized trials (RoB 2) [18] to assess the risk of bias in randomized controlled trials (RCTs). For observational studies, we predefined the following factors as confounding variables based on the literature: (1) preoperative level of physical activity [19], (2) American Society of Anesthesiologists physical status classification [19-21], (3) performed procedure [22], (4) open versus laparoscopic approach [20,23-25], (5) duration of surgery [20,21,23], and (6) postoperative intensive care unit admission [20,23]. Two independent assessors (MF and CK) evaluated each study, and conflicts were resolved through consensus. We used R statistical software (version 4.1.1; R Foundation for Statistical Computing) and the R package *robvis* [26] for data visualization.

### Data Synthesis

Because of the heterogeneity of study populations, methods, and device models, the data were not amenable for a meta-analysis. We performed a qualitative synthesis by summarizing the findings in three themes: (1) device use, (2) metrics used to describe physical activity, and (3) clinical outcomes analyzed in association with physical activity. Observational studies and RCTs were organized separately, given the differences in study designs. The key findings of individual studies were summarized in tables by tabulating the following variables: type of surgery, patient characteristics, main predictor (observational studies), intervention and control (RCTs), and main findings.

## Results

### Literature Search

**Figure 1:**
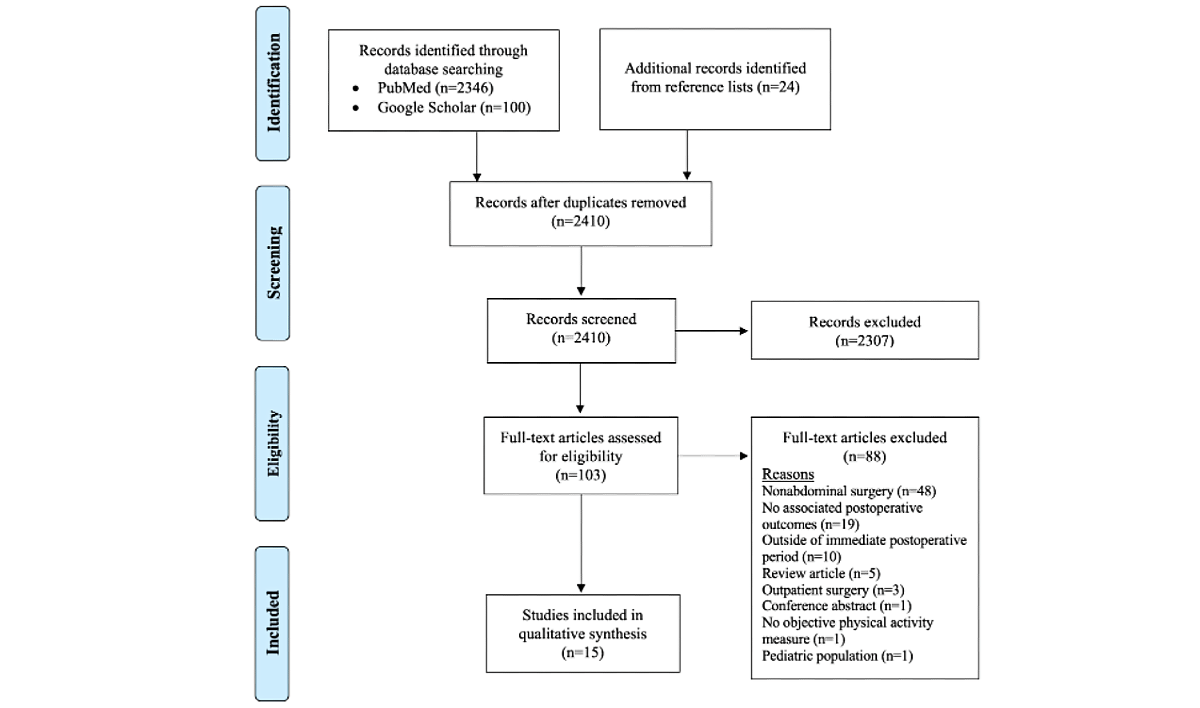
PRISMA (Preferred Reporting Items for Systematic Reviews and Meta-Analyses) flow diagram of literature search and study selection.

We identified 2470 articles: 2446 (99.03%) through the database searches and 24 (0.97%) from the review of reference lists. After screening the titles and abstracts of all 2470 articles, 103 (4.17%) underwent a full-text review. Of these 103 articles, 15 (14.6%) met our selection criteria [27-41]. The reasons for exclusion are detailed in Figure 1.

### Characteristics of the Included Studies

All articles were published between 2017 and 2021, except for the study by Browning et al [27], which was published in 2007. Of the 15 articles, 9 (60%) were observational studies [27-35], and 6 (40%) were RCTs [36-41]. The median sample sizes were 54 (IQR 50-94) for observational studies and 98 (IQR 64-107) for RCTs. Of the 15 articles, 13 (87%) studied the general surgery population, 1 (7%) was a study of patients who had undergone gynecologic surgery [40], and 1 (7%) included a mixed (abdominal, thoracic, gynecologic, and orthopedic) surgical population [30]. The results of individual studies are summarized in Tables 1-3.

**Table 1:** Summary of observational studies: physical activity predictors and associated outcomes.

Studies	Type of surgery (number of patients)	Patient characteristics	Main predictor	Main findings
Browning et al [27], 2007	Upper abdominal surgery (50)	Age: mean 61 (SD 12) years; BMI: mean 27.1 (SD 4.3) kg/m^2^; ASA^a^ classification I and II: 62% and ASA classification III: 38%; laparoscopic surgery: 0%; and LOS^b^: median 8 (IQR 3-121) days	Duration of uptime (standing or walking) during the first 4 postoperative days	A shorter uptime during the first 4 postoperative days was predictive of longer LOS (*R*^2^=0.50; *P*<.001); patients who developed pulmonary complications spent shorter uptime during the first 4 postoperative days
Low et al [28], 2018	Hyperthermic intraperitoneal chemotherapy with cytoreductive surgery (54)	Age: mean 57 (SD 11) years; BMI: mean 27.3 (SD 5.8) kg/m^2^; ASA classification II: 23% and ASA classification III and IV: 77%; laparoscopic surgery: 0%; and LOS: mean 12 (SD 7) days	Postoperative step count	Taking more steps during the inpatient recovery period predicted a lower risk of *30-day readmission* (OR^c^ 0.83, 95% CI 0.72-0.96; *P*=.01) for each additional 100 steps taken per day
Jonsson et al [29], 2018	Acute high-risk abdominal surgery (50)	Age: mean 61 (SD 17) years; BMI: mean 25.0 (SD 5.8) kg/m^2^; ASA classification I and II: 66% and ASA classification III and IV: 34%; laparoscopic surgery: 16%; and LOS: median 12 (IQR 7-22) days	Independent ambulation within the first operative week	Patients who achieved independent ambulation within the first postoperative week had fewer pulmonary complications than those who did not achieve independent ambulation (14% vs 53%, respectively; *P*=.01) and had a shorter LOS (8 days vs 22 days; *P*=.001)
Daskivich et al [30], 2019	Abdominal, thoracic, gynecologic, and orthopedic surgery (100)	Age: mean 53 (SD 18) years; BMI: mean 31 (SD 12) kg/m^2^; abdominal surgery: 79%; and LOS: median 4 (IQR 3-6) days	Step count on postoperative day 1	Higher step count on postoperative day 1 was associated with a lower probability of a prolonged LOS (OR 0.63, 95% CI 0.45-0.84; *P*=.003) for every additional 100 steps taken; no further reduction in LOS was observed beyond 1000 steps
Martin et al [31], 2020	Colorectal surgery (50)	Age: mean 59 (SD 18) years; BMI: mean 25.4 (SD 4.3) kg/m^2^; ASA classification I and II: 86% and ASA classification III and IV: 14%; minimally invasive surgery: 88%; and LOS: prolonged; >5 days	Postoperative step count during the first 3 postoperative days	Patients with postoperative complications took fewer steps during the first 3 postoperative days than those without complications (daily average: 1101, SD 2198 vs 1243, SD 1641 steps, respectively; *P*=.02); daily average step count was negatively correlated with LOS (*r*=−0.31; *P*=.03)
Nevo et al [32], 2021	Major abdominal surgery (91)	Age: mean 55 (SD 14) years; BMI: median 25.9 (IQR 20.9-30.9) kg/m^2^; ASA classification I and II: 59% and ASA classification III: 41%; laparoscopic surgery: 41%; and LOS: median 6 (IQR 4-8) days	Step count on postoperative day 2	Patients who took >1050 steps on postoperative day 2 had fewer postoperative complications (32% vs 71%; *P*<.05) and had shorter time to flatus (2.4 days vs 3.3 days; *P*<.01), time to bowel movement (1.8 days vs 3.2 days; *P*<.01), and LOS (5.4 days vs 8.8 days; *P*<.01), as well as lower readmission rate (*P*<.05)
Iida et al [33], 2021	Hepatectomy (147)	Age: >60 years; BMI: >20.0 kg/m^2^; laparoscopic surgery: 59%; and median LOS: 9, 14, and 12 days for upward slope, bell curve, and flat types, respectively	Physical activity trend patterns	Postoperative complications occurred in 4.5%, 76.9%, and 65.2% for upward slope, bell curve, and flat types, respectively (*P*<.001). Pneumonia was only observed among the flat type
Yi et al [34], 2021	Bowel surgery (37)	Age: mean 39 (SD 14) years; BMI: mean 27.7 (SD 8.3) kg/m^2^; ASA classification II: 70% and ASA classification III: 30%; laparoscopic or robotic surgery: 84%; and LOS: 6 days	Postoperative step count	Postoperative step count was not associated with LOS
Kane et al [35], 2021	Colorectal surgery (94)	Age: median 55.5 (IQR 25.5-61.5) years^d^ and median 58.0 (IQR 42.0-65.0) years^e^; BMI: median 29.1 (IQR 23.0-36.1) kg/m^2^^d^ and median 28.5 (IQR 23.5-30.4) kg/m^2^^e^; ASA classification II: 53% and ASA classification III: 47%; laparoscopic surgery: 46%; and LOS: median 3 (IQR 2-4) days	Step count on the day of discharge	A higher step count on the day of discharge was associated with a lower 30-day readmission risk; each 10% increase in return to preoperative baseline steps was associated with a 40% decrease in risk of 30-day readmission (OR 0.60, 95% CI 0.39-0.91; *P*=.02)

^a^ASA: American Society of Anesthesiologists.

^b^LOS: length of stay.

^c^OR: odds ratio.

^d^Patients who were readmitted.

^e^Patients who were not readmitted.

**Table 2:** Summary of randomized controlled trials (patient population).

Studies	Type of surgery	Patient characteristics	
		Control	Intervention
Fiore et al [36], 2017	Colorectal surgery	n=49; age: median 63 (IQR 48-72) years; BMI: median 26.2 (IQR 23.1-30.7) kg/m^2^; ASA^a^ classification I and II: 84%; laparoscopic surgery: 80%; and LOS^b^: median 3 (IQR 3-4) days	n=50; age: median 65 (IQR 51-71) years; BMI: median 26.6 (24.0-29.2) kg/m^2^; ASA classification: I and II: 86%; laparoscopic surgery: 82%; and LOS: median 4 (IQR 2-4) days
Ni et al [37], 2018	Hepatectomy	n=60; age: mean 49 (SD 15) years; and LOS: mean 7.7 (SD 2.1) days	n=59; age: mean 51 (SD 17) years; and LOS: mean 6.6 (SD 2.3) days
Wolk et al [38], 2019	Major visceral surgery	n=54; age: mean 57 years; BMI: mean 26.1 kg/m^2^; ASA classification I and II: 65% and ASA classification III: 31%; laparoscopic surgery: 50%; and LOS: 12 days	n=56; age: mean 60 years; BMI: mean 25.7 kg/m^2^; ASA classification I and II: 59% and ASA classification III: 39%; laparoscopic surgery: 52%; and LOS: 13 days
Waller et al [39], 2021	Elective colorectal surgery	n=23; age: 54 (SD 18) years; laparoscopic surgery: 52%	n=20; age: 54 (SD 13) years; laparoscopic surgery: 60%
No et al [40], 2021	Gynecologic midline laparotomy	n=28; age: 55 (SD 12) years; ASA classification I and II: 93% and ASA classification III: 7%; and LOS: median 6 (range 4-26) days	n=35; age: 53 (SD 10) years; ASA classification I and II: 100%; and LOS: median 7 (range 4-58) days
Steffens et al [41], 2021	Liver, gastric, and pancreatic cancer	n=49; age: median 64 (IQR 53-71) years; BMI: median 26.2 (IQR 21.9-29.2) kg/m^2^; laparoscopic surgery: 27%; and LOS: median 9 (IQR 6-15) days	n=47; age: median 65 (IQR 54-73) years; BMI: median 25.0 (22.5-29.7) kg/m^2^; laparoscopic surgery: 21%; and LOS: median 11 (IQR 7-17) days

^a^ASA: American Society of Anesthesiologists.

^b^LOS: length of stay.

**Table 3:** Summary of randomized controlled trials (interventions and main findings).

Studies	Control	Intervention	Main findings
Fiore et al [36], 2017	Usual care: preoperative instruction on early mobility, facilitation of postoperative mobilization by a nurse or nursing assistant, physiotherapy referral as needed, transfer to chair for 2 hours (day of surgery), and out of bed for at least 6 hours (from POD^a^ 1 until discharge)	Facilitated mobilization: in addition to usual care, physiotherapy education and assistance on mobilization (once on the day of surgery and 3 times daily from POD 1 until discharge), as well as a minimal 200-m walking target per session	More patients in the intervention arm got out of bed on the day of surgery and spent at least 6 hours out of bed on POD 1 and POD 2; step counts were at least 2-fold greater in the intervention arm on POD 1 and POD 2; primary and secondary outcomes were similar between the control and intervention groups (primary outcome: proportion of patients who returned to baseline 6MWT^b^ on postoperative week 4, 54% vs 51%; *P*=.58; secondary outcomes: median time to recovery of gastrointestinal function, 52.9 hours vs 46.6 hours; *P*=.60; median time to readiness to discharge, 3 days vs 3 days; *P*=.45; and complication rate, 48% vs 43%)
Ni et al [37], 2018	Usual care: bed activities (POD 1 and POD 2), bedside standing (POD 3), and ambulation (POD 4 and POD 5)	Early ambulation: preoperative education on early mobility, passive range of motion activities (the day of surgery), sit at the edge of the bed (POD 1), ambulate 2 to 3 times (POD 2), and ambulate more than 5 times (POD 3)	Patients in the intervention group took more steps from POD 2 to POD 5 and had shorter time to flatus (2.3 days vs 3.1 days; *P*=.04) and shorter LOS^c^ (6.6 days vs 7.7 days; *P*=.01); rates of postoperative complications were similar between the groups
Wolk et al [38], 2019	Usual care: ERAS^d^ protocol and blinded activity tracker wristband with no feedback or step count targets	Daily activity feedback: ERAS protocol and unblinded activity tracker wristband with daily activity feedback as well as predefined daily step count targets that progressed during the first 5 PODs	The intervention resulted in higher step counts during the first 5 PODs among patients who underwent laparoscopic surgery; postoperative complication rates and LOS were similar between the control and intervention groups for both patients who underwent open surgery and those who underwent laparoscopic surgery
Waller et al [39], 2021	Usual care: ERAS protocol, activity tracker without alarms, and daily ambulation target of 450 steps	Activity tracker with alarms: ERAS protocol, activity tracker with 5 daily alarms, and daily ambulation target of 600 steps	The intervention had no effect on postoperative step counts; postoperative outcomes (duration of ileus, incidences of pulmonary complications, venous thromboembolism, and LOS) were similar between the control and intervention groups
No et al [40], 2021	Usual care: encouragement for postoperative ambulation and blinded activity tracker with no feedback or step count targets	Activity tracker with feedback: in addition to usual care, patient self-monitoring of step counts and encouragement to meet daily targets until POD 5	Patients in the intervention arm took more steps on POD 4 and POD 5 and had higher percentage of recovery in steps from preoperative baseline than those in the control arm; however, the differences were not significant after adjusting for ASA^e^ classification (*P*=.90); outcomes were similar between the 2 groups (first flatus: 4 days vs 3 days; first diet: 3 days vs 3 days; ileus: 7% vs 9%; venous thromboembolism: none vs none; and LOS: 7 days vs 6 days)
Steffens et al [41], 2021	Usual care: preoperative counseling, daily physiotherapy sessions, and no activity tracker	Individualized target steps: in addition to usual care, individualized, progressive mobilization protocol with a study physiotherapist until hospital discharge	The intervention did not result in increased activity levels based on patient self-report (no objective activity data were collected from the control group); the patients in the intervention arm were more fatigued upon discharge; outcomes were similar between the intervention and control arms (postoperative complications: 45% vs 29%; *P*=.14; LOS: 11 days vs 9 days; *P*=.15; discharge home: 94% vs 92%; *P*=.99; and 30-day readmission: 17% vs 12%; *P*=.57)

^a^POD: postoperative day.

^b^6MWT: 6-minute walk test.

^c^LOS: length of stay.

^d^ERAS: Enhanced Recovery After Surgery.

^e^ASA: American Society of Anesthesiologists.

### Quality Assessment

**Figure 2:**
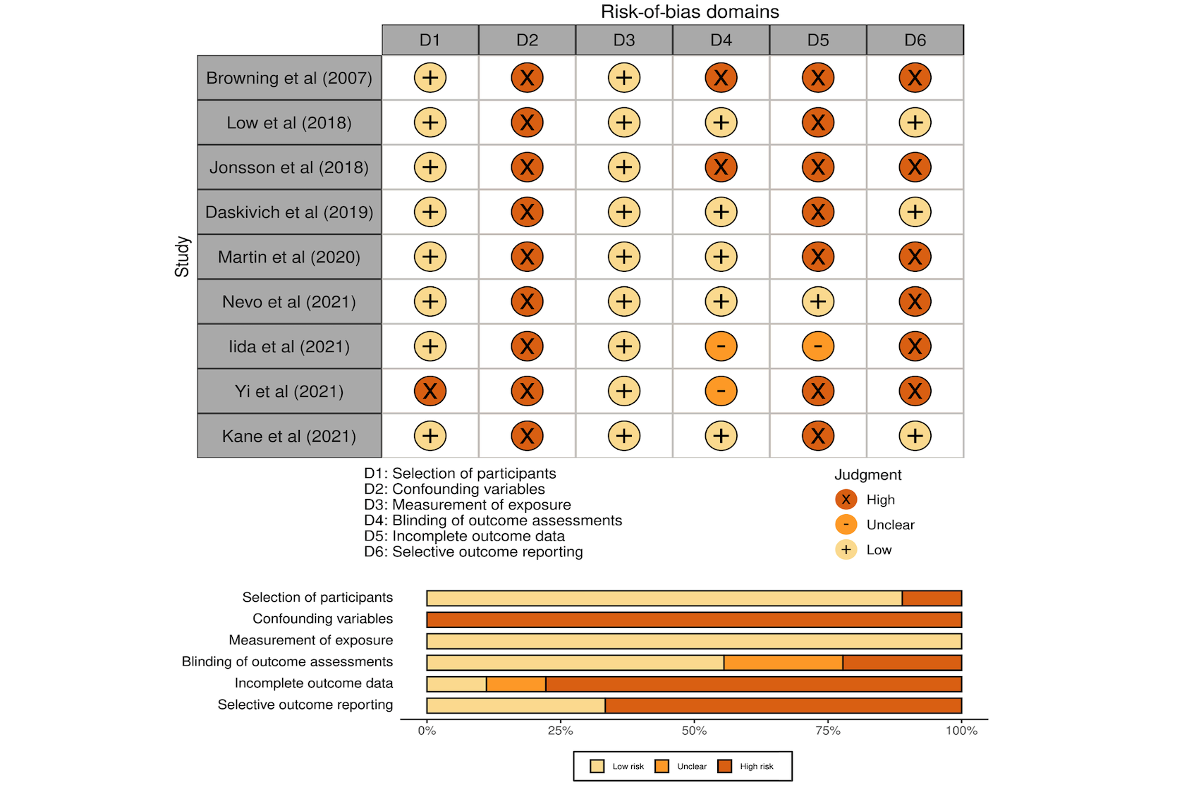
Quality assessment of observational studies using the risk-of-bias assessment tool for nonrandomized studies (RoBANS).

Figure 2 summarizes the risk-of-bias assessment for the observational studies (n=9), all of which used an accelerometer to collect physical activity data and were at low risk of bias for domain 3 (measurement of exposure). By contrast, none adequately controlled for confounders and were determined to be at high risk of bias for domain 2 (confounding variables). Of the 9 studies, only 3 (33%) clearly stated their study objectives [28,30,35]; the remaining 6 (67%) were exploratory, putting them at high risk of bias for domain 6 (selective outcome reporting) [27,29,31-34].

**Figure 3:**
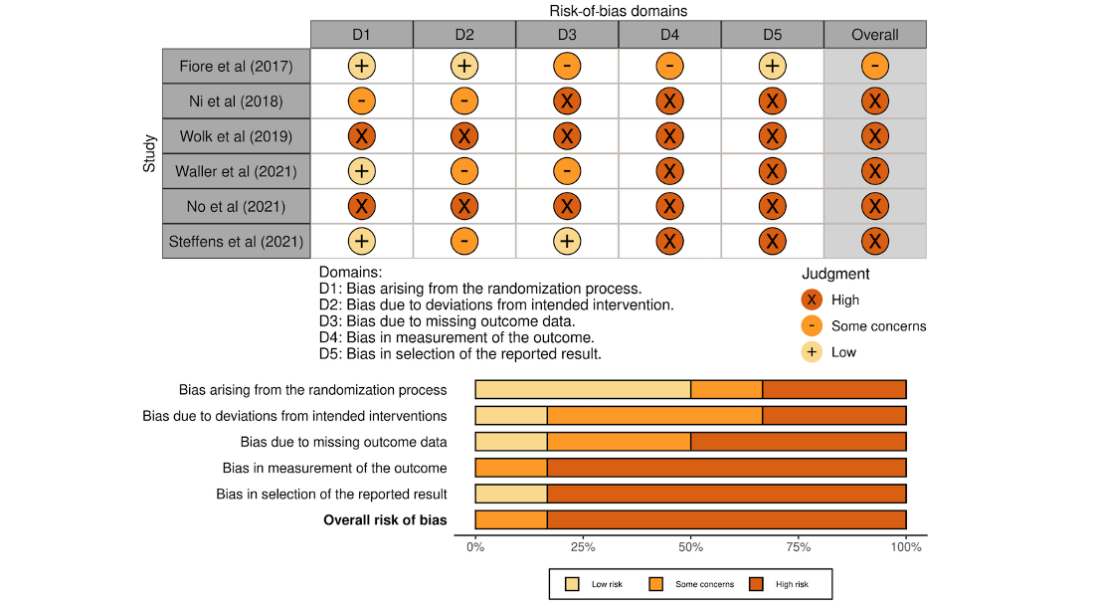
Quality assessment of randomized controlled trials using the revised Cochrane risk-of-bias tool for randomized trials (RoB 2).

Figure 3 shows the summary of the risk-of-bias assessment for the RCTs (n=6), all of which had at least some methodological concerns and were determined to have an overall high risk of bias, except for the study by Fiore et al [36], which was the most rigorous among these studies.

### Choice of Accelerometers

The device choice, use, and reported outcomes are summarized in Multimedia Appendix 3. Of the 15 studies, 3 (20%) used research-grade accelerometers [27,29,36], whereas the remaining 12 (80%) used consumer-grade devices [28,30-35,37-41]. Among the commercially available devices, the Fitbit series (Google LLC) was used most frequently (7/12, 58%) [28,30,34,35,37,39,41]. Of the 3 studies using research-grade accelerometers, only 1 (33%) study, which used the ActiGraph GT3X25 (ActiGraph LLC), described the accelerometry setup (such as sampling rate, filter, epoch, and analysis algorithm or software) [36].

### Device Wear Period

All studies used accelerometers during the acute inpatient period (per our inclusion criteria). Of the 15 studies, 12 (80%) applied the device within a day after surgery (n=7, 58%, studies on the day of surgery [30-32,35,36,38,39] and n=5, 42%, studies on postoperative day 1 or within 24 hours after surgery [27,29,33,37,40]). Of the 15 studies, 2 (13%) started the device wear after patients were transferred to the floor from the intensive care unit, which occurred on postoperative day 2 or 3 on average [28,41], and 1 (7%) did not report the timing of initiation [34]. The mean in-hospital wear duration was 5.2 (SD 2.0; range 3-10) days. Most of the studies (11/15, 73%) described continuous 24-hour device wear with or without brief interruptions for battery charging or patient showering [27-30,32-34,36,38,39,41], whereas others implied continuous measurement but did not explicitly describe it [31,35,37,40]. Of the 15 studies, 3 (20%) obtained preoperative baseline data, ranging from 2 to 30 days before surgery [31,35,40], and 1 (7%) followed patients after discharge until postoperative day 30 [35].

### Metrics Used to Describe Physical Activity

#### Step Count

Step count was the most commonly reported physical activity outcome, used in 12 (80%) of the 15 studies [28,30-32,34-41]. Of these 12 studies, 9 (75%) reported step count for each postoperative day [30,32,34-40]. Of these 9 studies, 3 (33%) averaged daily step counts over the entire postoperative study period, which ranged from 3 to 10 days [28,31,38], and 1 (33%) of these 3 studies also reported cumulative steps over the first 5 postoperative days [38].

Of the 12 studies that reported step count as the physical activity outcome, 3 (25%) measured preoperative step counts and also examined postoperative changes from baseline [31,35,40]. In the study by Martin et al [31], the average daily step counts decreased from 6444 (SD 4095) steps 5 days immediately before surgery to 1191 (SD 1864) steps 3 days after surgery (82% reduction). No et al [40] used preoperative step counts to calculate the percentage of recovery from baseline on postoperative days 4 and 5 to determine the efficacy of their activity-promoting intervention. Kane et al [35] calculated the percentage of return to preoperative baseline upon discharge and used it to predict 30-day readmission.

#### Activity Duration

Of the 15 studies, 2 (13%) observed that patients spent little time standing or walking (up to 0.6 hours per day) during the first week after surgery [27,29]. Fiore et al [36] measured the time spent out of bed (whether sitting or standing), which ranged from 0.6 to 0.9 hours on the day of surgery and from 6.7 to 10.3 hours between postoperative days 1 and 3. Wolk et al [38] reported an increase from 290 to 482 minutes of postoperative *activity time* per day, but *activity time* was not clearly defined.

#### Activity Trend

Many of the studies (11/15, 73%) presented daily activity trends using various outcome measures, including step counts [30-32,35-39], activity duration and sit-to-stand transitions [27,29], and energy expenditure [33]. These studies demonstrated that physical activity gradually increased after hitting the nadir immediately after surgery [27,29,30,32,33,35-39]. The studies by Daskivich et al [30] and Nevo et al [32] also showed that the recovery speed (measured in daily step counts) was different depending on the procedure type. Patients undergoing laparoscopic surgery had a more steady and faster recovery than those undergoing open surgery [33]. Nevo et al [32] and Iida et al [33] further analyzed how different recovery trajectories could inform the risks of developing postoperative complications (described in more detail in the *Surgical Complications* subsection).

#### Other Activity Metrics

Studies that used thigh-worn accelerometers reported daily numbers of sit-to-stand transitions [27,29]. Of the 15 studies, 2 (13%) tracked time-to-mobilization milestones such as sitting out of bed, standing, and walking [27,36]. Fiore et al [36] replicated the mobilization goals described in the 2005 ERAS guidelines (described in the *Introduction* section) [5].

### Clinical Outcomes Analyzed in Association With Physical Activity

#### Hospital Length of Stay

Of the 6 observational studies, 5 (83%) analyzed the impact of physical activity on hospital length of stay and found that being more active during the immediate postoperative period was associated with a shorter length of stay [27,29-32]; for example, in 1 (20%) of these 5 studies, every additional 100 steps up to 1000 steps on postoperative day 1 was associated with a shorter length of stay (odds ratio 0.63, 95% CI 0.45-0.84; *P*=.003) [30]. In another study, patients who achieved >1050 steps on postoperative day 2 had a shorter stay in the hospital than those who did not achieve that milestone (5.4 days vs 8.8 days, respectively; *P*<.01) [32].

By contrast, only 1 (17%) of the 6 RCTs demonstrated a reduction in hospital length of stay from interventions to enhance postoperative mobilization [37]. However, in 4 (67%) of the 6 RCTs, physical activity performances were similar between the control and the intervention arms [38-41]. In the study by Fiore et al [36], patients in the facilitated mobilization group spent more time out of bed and took twice as many steps on postoperative days 1 and 2 but had hospital lengths of stay similar to those of the patients in the control group.

#### Surgical Complications

Of the 9 observational studies, 3 (33%) tracked surgical complications and identified postoperative activity as a predictor of surgical complications, as defined by the Clavien-Dindo classification of surgical complications [31-33]. In these studies, higher step counts during the first 3 days [31,32] and a steady recovery trajectory during the first postoperative 7 days (*upward slope type*) [33] were predictive of fewer surgical complications. Conversely, Nevo et al [32] found that an acute drop in daily step count (a drop of >50% from the previous day and <500 steps) was strongly associated with severe complications (Clavien-Dindo grade ≥III; odds ratio 7.87, 95% CI 1.63-27.9; *P*=.007). Iida et al [33] described this pattern as a *bell curve type* and also noted a high complication rate among patients in this category (76.9%). In the same study, approximately one-third of the patients showed minimal progression in activity levels during the first 7 days after surgery (*flat type*), and 65.2% of them experienced complications [33].

Regarding the RCTs, mobility-enhancing interventions did not reduce surgical complications in any of the studies that evaluated these outcomes (4/6, 67%) [36-38,41], including the studies (2/4, 50%) that successfully increased physical activity in the intervention group compared with the control group [36,37].

#### Readmission

Of the 9 observational studies, 3 (33%) used inpatient activity to predict hospital readmission and found that higher step counts across the inpatient period (postoperative day 2 [32], inpatient average [28], and on the day of discharge [35]) were predictive of a lower likelihood of readmission after hospital discharge.

By contrast, of the 6 RCTS, 1 (17%) looked at 30-day readmission and did not find any difference between the intervention group and the control group (17% vs 12%, respectively; *P*=.57) [41].

#### Return of Gastrointestinal Function

Of the 9 observational studies, 1 (11%) found that patients who achieved a step count of >1050 on postoperative day 2 had a shorter time to first flatus (2.4 days vs 3.3 days; *P*<.01) and time to first bowel movement (3.2 days vs 4.9 days; *P*<.01) than those with fewer step counts [32].

Of the 6 RCTs, 4 (67%) evaluated the return of gastrointestinal function; only the study by Ni et al [37] demonstrated a shorter time to gastrointestinal recovery from a mobility-enhancing intervention (time to flatus: 2.3 vs 3.1 days; *P*=.04). The remaining RCTs (3/4, 75%), including the well-conducted study by Fiore et al [36], found no such effect from mobility-enhancing interventions [39,40].

#### Postoperative Pulmonary Complications

Of the 15 studies, 4 (27%; n=2, 50% of observational studies [27,29] and n=2, 50% of RCTs [36,39]) analyzed the effect of physical activity on postoperative pulmonary complications. Each study defined postoperative pulmonary complications differently, and the reported incidence rate ranged from 0% to 34%. In the observational studies, patients who developed pulmonary complications spent shorter times in upright positions (standing or walking) during the first 4 to 7 postoperative days than those who did not develop complications [27,29]. By contrast, the RCTs found no differences in pulmonary complications between the intervention and control groups. The overall incidence rates of pulmonary complications were very low in these RCTs (4% and 0%) [36,39].

#### Venous Thromboembolism

Of the 6 RCTS, 2 (33%) examined the incidence of venous thromboembolism [36,39]. Three patients developed venous thromboembolic complications in the study by Fiore et al [36], with no significant difference between the intervention and control groups. None of the 43 patients in the study by Waller et al [39] developed venous thromboembolism.

## Discussion

### Principal Findings

In this systematic review, we found 15 articles that used accelerometers to evaluate the effects of postoperative physical activity on outcomes after major abdominal surgery, with 14 (93%) published within the last 5 years. Although the observational studies (9/15, 60%) consistently showed that increased physical activity during the immediate postoperative period was associated with improved patient outcomes, only 1 (17%) of the 6 RCTs demonstrated that a mobility-enhancing intervention was beneficial compared with usual care. These findings confirm that physical activity is an important predictor of outcomes, but leave important questions unanswered—what is the optimal postoperative mobilization strategy or the dose of mobilization associated with better outcomes? Because of the high risks of bias, we could not synthesize specific mobility recommendations. However, our study illustrates how accelerometers can be a powerful tool for quantifying objective, continuous measures of physical behaviors in the hospital and provides guidance for future research to improve methodological rigors and study design.

We found from this systematic review that physical behaviors follow certain patterns after abdominal surgery. First, surgery causes a steep drop in physical activity from the preoperative baseline [31,35]. This effect is more significant after open abdominal surgery than after laparoscopic surgery [33] and varies by procedure type [30]. Second, the recovery of physical activity is slow, often requiring >1 month to return to baseline [30,33,35,39]. The recovery speed is also different, depending on the procedure [30-32], which is consistent with previous literature [24,25]. In the observational studies (n=9), increased physical activity during the immediate postoperative period was associated with improved clinical outcomes regarding surgical complications, return of gastrointestinal function, postoperative pulmonary complications, hospital length of stay, and hospital readmission [27-35]. These findings suggest that physical behaviors are important predictors of outcomes. In more practical terms, clinicians could use certain physical behaviors to predict or identify patients at risk for adverse outcomes after surgery.

Notably, 4 (67%) of the 6 mobility-enhancing interventions used in the RCTs did not increase postoperative mobilization compared with usual care [38-41]. The mobility-enhancing interventions ranged from step count feedback with encouragement to designated study physiotherapists assisting patients to achieve set mobility milestones. We speculate several reasons why many of these interventions (4/6, 67%) did not enhance mobility performances beyond usual care: (1) the selected interventions were simply ineffective, (2) the selected activity measure (step count was the most commonly used) was not sensitive enough to detect changes in mobility performances, and (3) mobility performances were nonmodifiable. Furthermore, the RCTs (2/6, 33%) that successfully enhanced physical activity showed conflicting effects on clinical outcomes. In the study by Ni et al [37], patients in the intervention arm achieved higher step counts from postoperative days 2 to 5 and had a faster return of gastrointestinal function and shorter hospital length of stay. By contrast, in the study by Fiore et al [36], more patients in the intervention arm were out of bed (sitting or standing) from the day of surgery through postoperative day 2 and took more steps on postoperative days 1 and 2 but had similar outcomes on return of gastrointestinal function and hospital length of stay.

Several factors could explain why postoperative mobilization had little effect on clinical outcomes when studied prospectively in the RCTs. First, the sample sizes of these RCTs were relatively small (median 98, IQR 64-107). Therefore, they could have lacked the statistical power to detect differences in clinical outcomes. Second, postoperative physical activity may be a prognostic indicator of outcomes rather than a modifiable factor. This theory is plausible, given that the factors associated with reduced postoperative mobilization and worse clinical outcomes often overlap, such as preoperative physical activity level [19-21], open versus minimally invasive approach [20,23-25], and duration of surgery [20,21,23]. Third, it is possible that the achieved differences in mobilization dosage (such as timing, type, duration, frequency, and intensity) [10,11] were not significant enough to affect clinical outcomes. Fourth, routine care that involves basic mobility may be sufficient to prevent immobility harm. Fifth and last, the effects of specific physical activity measures on postoperative outcomes remain unknown [8]; for example, it is unclear whether sitting out of bed (static positioning) is as effective as standing and walking (active mobility) in improving clinical outcomes. Thus, the choice of reported mobility metrics could have affected the researchers’ ability to detect clinically meaningful differences in activity exposures.

The particularly well-conducted study by Fiore et al [36] is worth special attention. The authors defined physical activity as “out of bed at all on the day of surgery and out of bed for at least 6 hours on postoperative day 1-3,” which directly reflects the recommendation described in the original ERAS guidelines [5]. This RCT found no benefit from the authors’ facilitated mobilization intervention, including the 6-minute walk test at 4 weeks (primary outcome), time to gastrointestinal recovery, time to readiness to discharge, length of stay, and 30-day complications. The negative result may be partly due to patient selection because 80% (80/99) of the study participants received laparoscopic surgery. Laparoscopic surgeries have been shown to expedite recovery [24,25,38], and treatment effects from mobility-enhancing interventions may be less pronounced in patients undergoing laparoscopic surgeries than in those undergoing open surgeries, especially in environments with optimal usual care. In the case of the study by Fiore et al [36], patients in the usual care group reached activity levels similar to those reached by patients in the intervention arm by postoperative day 3.

We found that most of the studies (12/15, 80%) used consumer-grade accelerometers to characterize physical behaviors. Commercially available devices have appealing features such as patient familiarity, user-friendly interfaces, and fashionable designs, all of which could improve wear compliance. In addition, measures such as step count are intuitive and easy to interpret among many users. However, consumer-grade accelerometers are different from research-grade accelerometers in that they use proprietary algorithms to compute and report physical behavior measures such as step count and energy expenditure. Furthermore, they do not give researchers access to accelerometer settings such as filter, sampling rate, epochs, and software algorithm. As patients who are hospitalized are distinct from the free-living population in that they spend most of their wakeful time sedentary or in bed [42-44], walk significantly slower, and may hold on to an intravenous pole or an assistive device when ambulating [43], researchers may benefit from using research-grade accelerometers because of their flexibility in terms of data collection and analysis [45].

Importantly, most validation studies of accelerometer devices are derived from laboratory and free-living conditions. These studies show that measurements can vary substantially by device manufacturer [46-50], wear location [47,49-52], and data-processing algorithm [46,48]. For step count, the most commonly reported physical activity outcome in our review (12/15, 80%), the discrepancy in measurement can be as much as 120%, depending on the device and wear location [50]. Moreover, the study population and setting can affect device accuracy; for example, older adults, who tend to walk slower than younger adults, walked at a speed of 0.74 meters per second as outpatients but recorded a speed of 0.46 meters per second as inpatients [42]. One study found 20% absolute percentage errors in step counts at a gait speed of 0.42 meters per second and 45% errors at an even slower pace [49]. Many accelerometers available in the market, including research-grade devices, still await validation in acute inpatient settings [42,53]. As is the case with laboratory biomarkers, digital biomarkers derived from biometric monitoring technologies require multistep validation before they can be applied reliably to a specific patient population and clinical setting [54]. It is critical to be mindful of these limitations when interpreting the results or conducting research using accelerometers because the reported outcomes, particularly step count, are not directly comparable [50-52].

### Strengths and Limitations

There are 2 major limitations related to the conduct of this systematic review. First, database searches were limited to PubMed and Google Scholar owing to our time constraints; therefore, we could have missed articles available in other databases. To supplement this, we used the reference lists of the included studies and related review articles to identify relevant studies. Second, the heterogeneity of study designs, patient populations, and accelerometer use made it difficult to compare study findings. To minimize the risk of bias resulting from data synthesis, we developed and followed a written protocol using rigorous systematic review processes.

### Future Directions

Overall, the quality of available evidence was poor, and we could not synthesize specific recommendations for postoperative mobilization. On the basis of the limitations we identified in the included studies, we recommend that researchers (1) select a patient population that is more likely to benefit from mobility-enhancing interventions (eg, patients undergoing open abdominal surgery rather than laparoscopic surgery and patients with frailty rather than those who are young, healthy, and fit); (2) clearly define and measure timing, type, duration, frequency, and intensity of a mobility-enhancing intervention to delineate the differences in mobility performances achieved by patients in different treatment groups; (3) measure all relevant data (such as patient, surgical, and postoperative factors) to control for confounders adequately; and (4) measure physical behaviors beyond step counts (such as static positioning and in-bed activities) because patients are highly sedentary after surgery [55,56], and step counts only capture snapshots of patients’ mobility status.

### Conclusions

In conclusion, although observational studies showed strong associations between postoperative physical activity and outcomes after major abdominal surgery, RCTs have not proven the benefit of mobility-enhancing interventions compared with usual care. To understand the optimal postoperative mobilization strategy or the impact of individual physical activity components such as timing, type, duration, frequency, and intensity, future accelerometer research would benefit from improved study designs, increased methodologic rigor, and more consistent reporting of accelerometer methods [57].

## Appendix

Multimedia Appendix 1 PRISMA (Preferred Reporting Items for Systematic Reviews and Meta-Analyses) checklist.

Multimedia Appendix 2 PubMed and Google Scholar search strategies.

Multimedia Appendix 3 Device choice, use, and reported outcomes.

## Abbreviations

ERAS: Enhanced Recovery After Surgery
MeSH: Medical Subject Headings
PRISMA: Preferred Reporting Items for Systematic Reviews and Meta-Analyses
RCT: randomized controlled trial
RoB 2: revised Cochrane risk-of-bias tool for randomized trials
RoBANS: risk-of-bias assessment tool for nonrandomized studies

## Data Availability

The data sets generated or analyzed during this study are available from the corresponding author on reasonable request.
