# Supplementary material for: Accelerometer-Measured Inpatient Physical Activity and Associated Outcomes After Major Abdominal Surgery: Systematic Review
Source: Interact J Med Res. 2023 May 15;12:e46629. doi: 10.2196/46629 (PMC10227699; doi:10.2196/46629)
Supplement: Multimedia Appendix 2 [file ijmr_v12i1e46629_app2.docx]

Multimedia Appendix 2-A. PubMed search strategy

|  | Search Query |
| --- | --- |
| #1 | "Accelerometry"[Mesh] OR Acceleromet* OR Actigraph* |
| #2 | "Fitness Trackers"[Mesh] OR "Fitness Track*" OR "Activity Track*" |
| #3 | "Wearable Electronic Devices"[Mesh] OR Wearable* |
| #4 | "Postoperative Period"[Mesh] OR "Postoperative Care"[Mesh] OR Postoperative[tiab] OR post operative[tiab] OR postsurgical*[tiab] OR post surgical* OR surger*[tiab] OR surgic*[tiab] |
| (#1 OR #2 OR #3) AND #4^a^ | ("Postoperative Period"[MeSH Terms] OR "Postoperative Care"[MeSH Terms] OR "Postoperative"[Title/Abstract] OR "post operative"[Title/Abstract] OR "postsurgical*"[Title/Abstract] OR ("post"[All Fields] AND "surgical*"[All Fields]) OR "surger*"[Title/Abstract] OR "surgic*"[Title/Abstract]) AND ("Accelerometry"[MeSH Terms] OR "acceleromet*"[All Fields] OR "actigraph*"[All Fields] OR ("Fitness Trackers"[MeSH Terms] OR "fitness track*"[All Fields] OR "activity track*"[All Fields]) OR ("Wearable Electronic Devices"[MeSH Terms] OR "wearable*"[All Fields])) |

^a^Final search strategy for PubMed

Multimedia Appendix 2-B. Google Scholar search strategy

|  | Search Query |
| --- | --- |
| #1 | "Accelerometry" OR "acceleromet*" OR "actigraph*" OR "Fitness Trackers" OR "fitness track*" OR "activity track*" OR "Wearable Electronic Devices" OR "wearable*" |
| #2 | Postoperative Period OR "Postoperative Care" OR "Postoperative" OR "post operative" OR "postsurgical*" OR ("post" AND "surgical*") OR "surger*" OR "surgic*" |
| #1 AND #2^b^ | ("Accelerometry" OR "acceleromet*" OR "actigraph*" OR "Fitness Trackers" OR "fitness track*" OR "activity track*" OR "Wearable Electronic Devices" OR "wearable*") AND (Postoperative Period OR "Postoperative Care" OR "Postoperative" OR "post operative" OR "postsurgical*" OR ("post" AND "surgical*") OR "surger*" OR "surgic*") |

^b^Final search strategy for Google Scholar
