# Supplementary material for: Accelerometer-Measured Inpatient Physical Activity and Associated Outcomes After Major Abdominal Surgery: Systematic Review
Source: Interact J Med Res. 2023 May 15;12:e46629. doi: 10.2196/46629 (PMC10227699; doi:10.2196/46629)
Supplement: Multimedia Appendix 3 [file ijmr_v12i1e46629_app3.docx]

Multimedia Appendix 3. Device choice, use, and reported outcomes


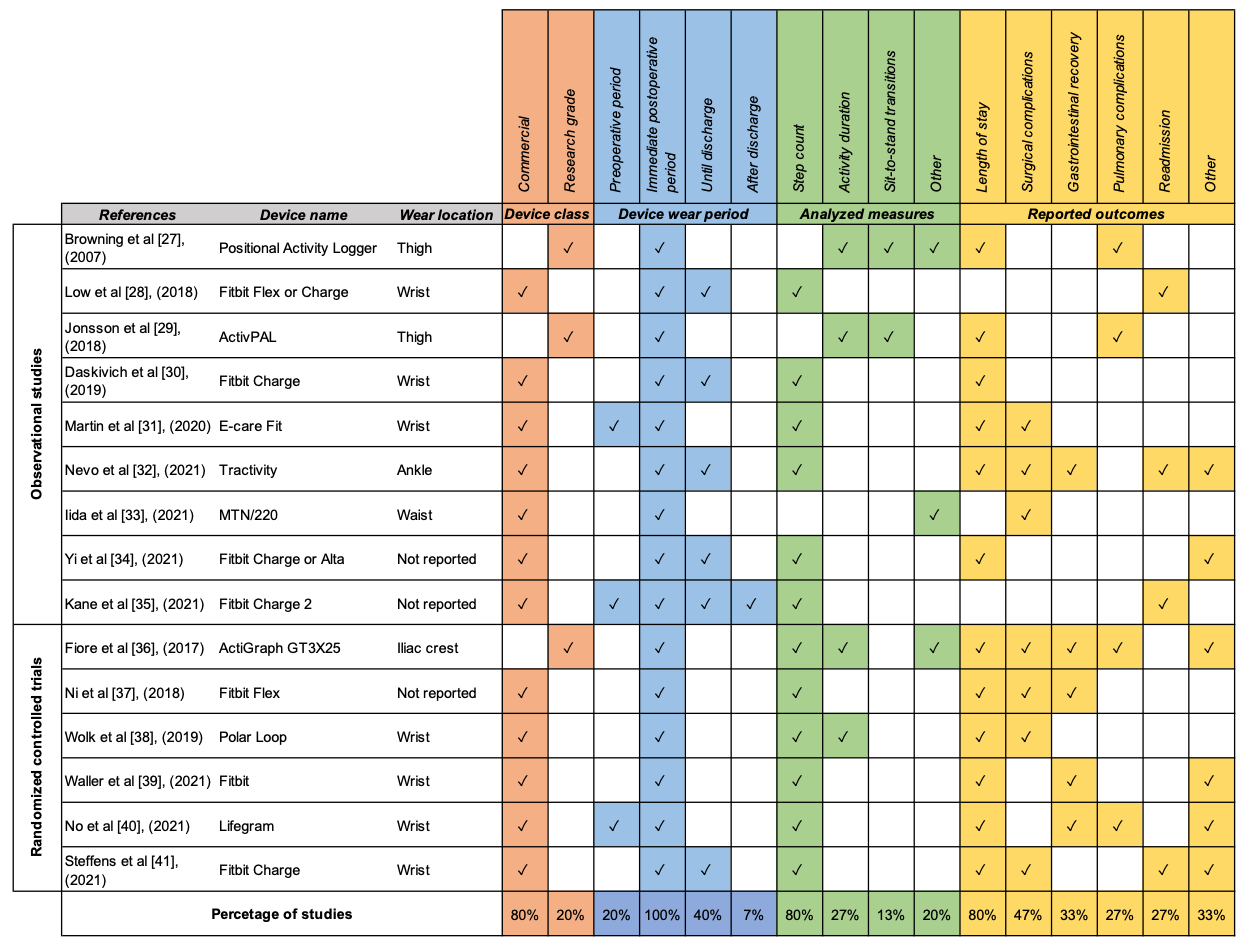


Other analyzed measures include time-to-mobilization milestones [27,36] and daily energy expenditure [33].

Other reported outcomes include discharge destination [41], time to readiness for discharge [36], venous thromboembolism [36,39], pain [40,41], postoperative nausea and vomiting [32,34], and fatigue [40,41].
